# Fabrication and Characterization of Transparent and Uniform Cellulose/Polyethylene Composite Films from Used Disposable Paper Cups by the “One-Pot Method”

**DOI:** 10.3390/polym14061070

**Published:** 2022-03-08

**Authors:** Lixiang Wang, Qiwen Zhou, Xiaoqian Ji, Jianfeng Peng, Haq Nawaz, Guangmei Xia, Xingxiang Ji, Jinming Zhang, Jun Zhang

**Affiliations:** 1 Key Laboratory of Paper Science and Technology of Ministry of Education, Faculty of Light Industry, Qilu University of Technology, Shandong Academy of Sciences, Jinan 250353, China; 15726110037@163.com (L.W.); biliqwzhou@163.com (Q.Z.); jxq20021220@163.com (X.J.); pjf1966614429@163.com (J.P.); 2 Beijing National Laboratory for Molecular Sciences, CAS Key Laboratory of Engineering Plastics, Institute of Chemistry, Chinese Academy of Sciences (CAS), Beijing 100190, China; haqnawaz@bjfu.edu.cn (H.N.); jzhang@iccas.ac.cn (J.Z.)

**Keywords:** disposable paper cups, recycle, cellulose, polyethylene composite films

## Abstract

Disposable paper cups are usually composed of high-grade paper board and an inner polyethylene coatings and are extensively used in daily life. However, most disposable paper cups are only used for a short time and then incinerated or accumulated in landfill at the end of their service due to the difficulty in separating the components, leading to a serious threat to our ecosystem. Therefore, developing a facile and green method to recycle and reuse disposable paper cups is vital. By using ionic liquid 1-allyl-3-methylimidazolium chloride (AmimCl) as a solvent, transparent and homogenous cellulose/polyethylene composite films were successfully prepared from used bamboo-based disposable paper cups through the “one-pot method”, without any pre-treatment. It was found that there was a transformation of cellulose I to II after the dissolution and regeneration processes, and the crystallinity degree of the regenerated cellulose-based materials decreased significantly, resulting in a change in thermal properties. Meanwhile, compared to traditional pure cellulose films, the composite films possessed good UV-shielding properties and hydrophobicity. Moreover, they also displayed good mechanical properties. Additionally, the size of the ground PE coatings displayed obvious effects on the structures and properties of the composite films, where the CPE100 (sieved with 100–200 mesh) possessed the most homogeneous texture and the highest tensile strength (82 Mpa), higher than that of commercial polyethylene film (9–12 MPa), showing superiority as packaging or wrapping materials. Consequently, the goals to fabricate uniform cellulose/polyethylene composite films and valorize the solid waste from disposable paper cups were simultaneously achieved by a facile and green “one-pot method”.

## 1. Introduction

With the development of the economy and the continuous growth of the global population, disposable products are widely used and promoted [1,2,3,4]. Disposable paper cups (DPCs) have many advantages, such as relative sanitation, low price, convenient use, and huge output, and they are extensively employed in many families, enterprises, and public places [5]. According to statistics, the global consumption of disposable paper cups has exceeded 220 billion and reached 32 per capita in 2018 [6]. Additionally, it is reported that the amount of annually consumed disposable paper cups in the UK and China has reached more than 2.5 billion and 10 billion, respectively [6,7]. It is known that paper cups consist of approximately 95% high-degree cellulose paper board, indicating that a large quantity of trees have been cut down for their manufacture, leading to ecosystem destruction [8,9,10]. Meanwhile, the production process of paper cups consumes large quantities of natural resources and discharges vast volumes of carbon dioxide, resulting in a significant waste of energy and an increased greenhouse effect [11,12,13]. Therefore, recycling and reusing disposable paper cups is important and meaningful, and is essential for the protection of our ecosystem and to make full use of natural resources. As their name suggested, it is difficult to reuse disposable paper cups, and they are usually abandoned as waste after use because they are employed by many people for occasional, brief use [10,14]. It was found that the United States throw away 50 billion paper cups each year [6,7]. Meanwhile, more than 7000 t of non-recyclable disposable paper cup waste has been generated due to Australia’s obsession with coffee [10]. Consequently, large quantities of used disposable paper cups (UDPCs) are incinerated or landfilled in the environment [6,12,15]. Nevertheless, these two processes are recognized as ecologically unfriendly due to the emission of toxic gases or the un-degradability of the inner thin polyethylene (PE) coatings [5,16,17]. It has been reported that non-degradable petrochemical materials have migrated from inland to the ocean, where they accumulate. Unfortunately, some of them have amassed in the food chain, posing a serious threat to animals and human [9]. Furthermore, it will take many years for them to decay, which poses a potential disaster for the natural environment. Hence, waste recovery is urgent and meaningful, and developing an environmentally-friendly processes to recycle and reuse these used paper cups is vital.

Up to now, several attempts have been made to recycle and reuse UDPCs [17,18,19,20]. After peeling off the PE coatings from used disposable paper cups, Chen et al. [19] and Nagarajan et al. [18] fabricated cellulose derivative carboxymethylcellulose and cellulose nanomaterials. Generally, disposable paper cups are composed of 5% inner polyethylene (PE) coatings to prevent leakage [20]. However, the outer cellulose paper board and the inner PE coatings are bonded tightly, and separating them economically and thoroughly is difficult. Hence, Mitchell et al. [21] prepared novel paper–plastic composites (PPCs) by employing un-peeled shredded paper cups and polypropylene as additive and matrix, respectively. Meanwhile, Zhao et al. prepared graphene sheets directly from disposable paper cups by using Fe^2+^ as a catalyst [22], and P-carbon was also achieved by using wastepaper cups as the raw material [17]. Additionally, vermicomposting is an eco-friendly method to dispose of used paper cups, but it takes a long time for paper cups to degrade [8,11]. As discussed, most processes have the disadvantages of being environmentally unfriendly or energy- or time-intensive, requiring harsh conditions, thus limiting the simultaneous up-cycling of both paper board and PE coatings within one process. Therefore, investigations into exploiting facile and green methods to completely utilize and valorize used disposable paper cups within one process need to be initiated.

With the depletion of fossil resources and global catastrophic climate change, biomass resources with renewability, biodegradability, and eco-friendliness have gained extensive attention over the last decade [23,24,25]. As the most abundant component in biomass, cellulose is ubiquitous on earth and has been widely transformed into various functional materials and applied in packaging and wrapping [26,27,28], fluorescent smart materials [29], energy storage materials [30,31], and so on. However, high-cost wood pulp and cotton pulp are usually two major sources of cellulose, limiting the development of renewable resources [32,33]. By contrast, low-coat solid wastes such as used disposable paper cups can be a good resource for cellulose.

With the development of science and technology, it is known that materials with single components cannot always meet social needs, and composite materials now play an important role in our daily life [34,35]. Bio-composites, natural fiber-reinforced polymer composites, are now recognized as being significant composite competitor materials to conventional composite due to their attractive features, which include being lightweight, having high strength, and possessing a specific stiffness, and they have been widely investigated and used in various construction and building fields [36,37]. However, more efforts should be made in developing new bio-composites and optimizing these materials for new applications [35]. Generally, cellulose cannot be processed by the melting method or dissolved in most organic solvents because of their copious hydrogen bonds [38,39,40]. Fortunately, room ionic liquids (ILs) have been proven to show superior dissolving capacity for cellulose [39,40,41]. In our previous studies, by using 1-allyl-3-methylimidazolium chloride (AmimCl) ionic liquid, solid wastes such as waste newspapers [32], cotton textile wastes [27,42], corrugated cartons [43], and wood-based disposable paper cups [44] have been used as raw materials to obtain value-added cellulose materials. In this work, transparent and uniform cellulose/polyethylene composite films with good mechanical properties were successfully fabricated from used bamboo-based disposable paper cups by a “one-pot method”, without any pretreatment. In this current work, compared to the previous study, used disposable paper cups can be fully utilized and valorized within one process by a facile and green method, where the preparation process is environment-friendly and conducted in mild conditions [42]. Meanwhile, the structures and properties of the cellulose/polyethylene composite films were investigated by a series of characterizations. The results showed that the cellulose/polyethylene composite films had good hydrophobicity, UV-shielding, and mechanical and thermal properties, thus displaying great potential to be used in the packaging and wrapping fields.

## 2. Materials and Methods

### 2.1. Materials and Reagents

The high degree cotton pulps (CPs) and ionic liquid (1-allyl-3-methylimidazole chloride, AmimCl, Shandong, China) were kindly supplied by Shandong Henglian New Materials Co., Ltd. The degree of polymerization of CPs was 530 and AmimCl was synthesized by the method reported in our previous work [40]. Both bamboo-based disposable paper cups and commercial polyethylene (PE) film were bought from Jinan Hengyou New Material Technology Co., Ltd. All materials were dried in a vacuum oven at 80 °C for 48 h before use. The disposable paper cups containing 95% bamboo paper board and 5% PE coatings had only been used once for hot drinks. Deionized water was self-provided in the laboratory by ultra-pure water equipment.

### 2.2. Dissolution of UDPCs and Fabrication of Cellulose/Polyethylene Composite Films

**Figure 1:**
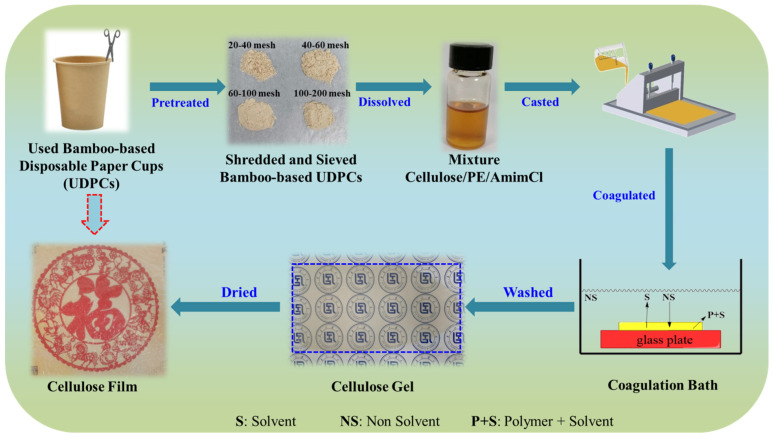
The processes for the dissolution of used bamboo-based disposable paper cups and the preparation of cellulose/polyethylene composite gels and films.

The processes for the dissolution of used bamboo-based disposable paper cups (UDPCs) and the preparation of cellulose/polyethylene composite gels and films are briefly depicted in Figure 1. Firstly, the used bamboo-based disposable paper cups (UDPCs) were ground into powder using a grinder and then sieved through four different standard mesh screens before use: 20–40 mesh (UDPCs 20), 40–60 mesh (UDPCs 40), 60–100 mesh (UDPCs 60), 100–200 mesh (UDPCs 100). Subsequently, 1.0 g of UDPCs and 49 g of AmimCl ionic liquid were added into a 100 mL three-necked flask, and the mixtures were mechanically stirring at 660 r/min until homogeneous solutions was obtained after 4 h at 80 °C. Finally, four kinds of cellulose/PE/AmimCl solution mixtures were obtained. The four resultant solution mixtures were then cast onto a glass plate to form a 1 mm thick liquid film controlled by a film spreader. The glass plate with 1 mm thick liquid film was then immediately placed into a deionized water coagulating bath to form cellulose/PE composite gels by the sol-gel method. To completely dispose of AmimCl, the water coagulating baths were changed at least five times until no Cl^−^ anion was detected by silver nitrate titration. Four kinds of brown and transparent gel were fabricated, and they were named Gel-CPE20, Gel-CPE40, Gel-CPE60, and Gel-CPE100. These four gels were dried at 98 °C for 12 min using a Kessel paper dryer to obtain cellulose/PE composite films (named CPE20, CPE40, CPE60, and CPE100). Because ionic liquids can be recycled completely and reused to dissolve cellulose again, the valorization of used bamboo-based disposable paper cups to prepare cellulose composite films via AmimCl is a relatively green process [42].

Additionally, cellulose gel (CP-gel) and cellulose film (CP-film) prepared from high-grade cotton pulps were also obtained under the same conditions as discussed above for comparison.

### 2.3. Characterization

#### 2.3.1. The Chemical Components in UDPCs

To assess the mass fraction of lignin, hemicelluloses, and cellulose in UDPCs, the GB/T 2677.8-1994, GB/T 10337-1989, GB/T 2677.10-1995, and Nitric acid-ethanol methods were employed, where the mass fractions of lignin, hemicelluloses, and cellulose were 12.53%, 13.74%, and 69.68%, respectively.

#### 2.3.2. The Degree of Polymerization (DP) of Cellulose in UDPCs

The degree of polymerization (DP) of cellulose in UDPCs was characterized according to the Standard Test Method for Intrinsic Viscosity of Cellulose (ASTM D795-13), where Ubbelodhe viscometry and cupriethylenediamine solution were employed, and the DP of cellulose in UDPCs was approximately 304.

#### 2.3.3. Polarized Optical Microscopy of UDPCs/AmimCl Solution

To show the dissolution process of UDPCs in AmimCl, a polarizing microscope (PM6000, Jiangnan Yongxin, Nanjing, China), purchased from Jiangnan Yongxin Optical Co., Ltd., was employed. The solution mixture was dropped onto the clean glass slide and sandwiched with a clean cover slip. The dissolution state of the UDPCs/AmimCl mixture was observed, and the pictures were recorded at 0 h, 0.5 h, 3 h, and 4 h.

#### 2.3.4. Ultraviolet and Visible (UV-Vis) Spectra of the Cellulose/Polyethylene Composite Films

The UV-Vis spectra of cellulose/PE composite films were recorded by Ultraviolet spectrophotometer (UV 2600, Shimadzu, Tokyo, Japan), in which the wavelength ranged from 200 nm to 800 nm and both the transmittance and absorbance modes were adopted.

#### 2.3.5. The Surface Hydrophilicity of the Cellulose/Polyethylene Composite Films

An OCA 50 machine (Dataphysics, Filderstadt, Germany) was used to characterize the surface hydrophilicity of the cellulose/PE composite films, where seven or eight spots were written for each film, and the average value was displayed. The water contact angles of cellulose/polyethylene composite films CPE20, CPE40, CPE60, and CPE100 were recorded.

#### 2.3.6. Wide-Angle X-ray Diffraction (WAXD) of the CPs, UDPCs, Commercial PE film, and Cellulose/Polyethylene Composite Films

The X-ray diffraction spectra of the raw materials and the composite films were recorded via X-ray diffractometer (D8 AD-VANCE, Bruker, Rheinstetten, Germany), where 40 kV, 40 mA, and CuKa Radiation (λ = 1.5406 Å) were adopted. Additionally, the scan speed and 2*θ* span were set at 8°/min and 5–60°, respectively.

#### 2.3.7. Fourier Transform Infrared (FTIR) Spectra of the CPs, UDPCs, Commercial PE film, and Cellulose/Polyethylene Composite Films

The chemical structures of the CPs, UDPCs (the PE side and after peeling off PE coatings), commercial PE film, and cellulose/PE composite films were investigated by attenuated total reflectance Fourier transform infrared spectrometer (ATR-FTIR ALPHA, Bruker, Rheinstetten, Germany), in which the resolutions were 32 scans and 4 cm^−1^, and three spots were assessed. The Germanium (Ge) crystal had to be washed between samples by ethanol. Finally, OPUS software was used to analyze the results.

#### 2.3.8. Mechanical Tests of the Cellulose/polyethylene Composite Films

According to the ASTM D-882 standard, the mechanical properties of the cellulose/PE composite films were measured by TA.XT Plus C texture Analyzer (StableMicroSystem, Surrey, UK), where the films were cut into samples 1 cm in width and 5 cm in length. Meanwhile, the drawing speed and gauge length were set at 2 cm and 5.0 mm/min, respectively. At least five strips were measured for each film, and then the average value was displayed.

#### 2.3.9. Thermogravimetric Analysis (TGA) of the CPs, UDPCs, Commercial PE Film, and Cellulose/Polyethylene Composite Films

The thermal decomposition behavior of the CPs, UDPCs, commercial PE film, and cellulose/polyethylene composite films was investigated using a thermogravimetric analyzer (TA Q50, New Castle, DE, USA). There was a ceramic pan inside the furnace of the TA Q50, which had a precision balance. The films needed to be cut into small pieces, and approximately 5 mg of the samples was placed in the crucible pot. The test was conducted at a heating rate of 10 °C/min in nitrogen atmosphere, where the temperature ranged from 50 °C to 800 °C.

#### 2.3.10. Morphology of Cellulose/Polyethylene Composite Films

The morphology pictures of cellulose/polyethylene composite films were characterized using a scanning electron microscopy (SEM, EM-30 Plus, COXEM, Daejeon, Korea). To obtain the cross-section images of the composites films, the sample had to be quenched in liquid nitrogen. All samples were sputter-coated with platinum before testing.

## 3. Results

### 3.1. Dissolution of Bamboo-Based Used Disposable Paper Cups

Rogers et al. [39] reported that ionic liquids displayed excellent dissolving ability for cellulose. Subsequently, it was found that ionic liquids were also a good solvent for lignocellulose, and AmimCl showed the most effective capacity for dissolving lignocellulose [40,41]. As characterized, the bamboo-based paper board contained 12.53% lignin, 13.74% hemicellulose, and 69.68% cellulose. In this work, AmimCl was employed to dissolve the bamboo-based UDPCs.

**Figure 2:**
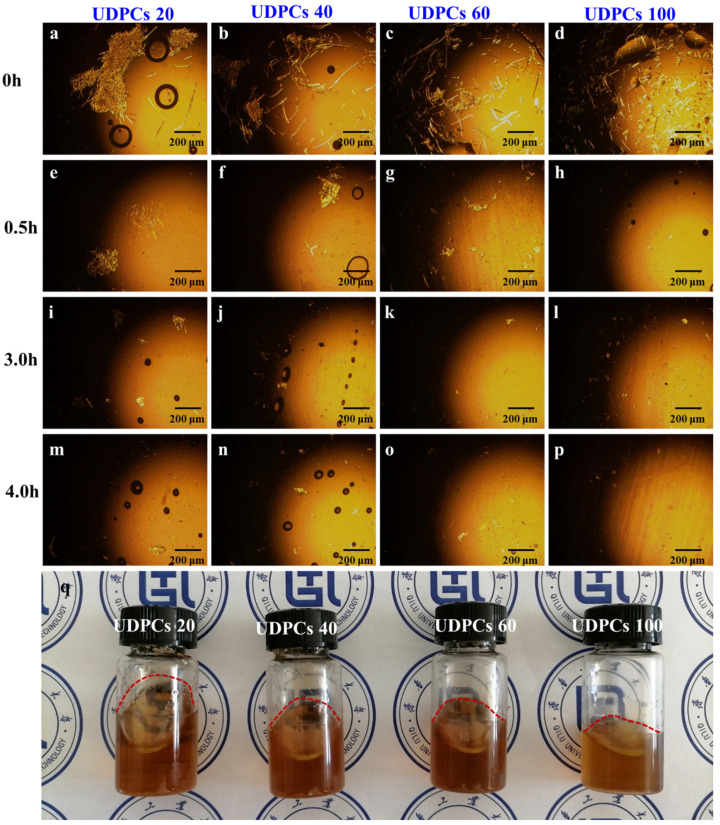
(**a**–**p**) POM micrographs of UDPCs with different sizes in AmimCl at 80 °C after (**a**–**d**) 0 h, (**e**–**h**) 0.5 h, (**i**–**l**) 3 h, and (**m**–**p**) 4 h. (**q**) Digital picture of cellulose/PE/AmimCl solution mixtures after 4 h.

As displayed in Figure 2a–p, the dissolution process of UDPCs with four different sizes in AmimCl at 0 h, 0.5 h, 3 h, and 4 h was recorded. It is obvious that UDPCs were composed of paper–plastic complex and copious microfibers. Meanwhile, the diameters of the microfibers ranged from 10 to 50 μm [32]. The flexible PE coatings can be ground into pieces with the brittle paper board because they were tightly bonded. It can be seen that the size of the paper–plastic complex in UDPCs 20 (sieved with 20–40 mesh) solution was larger than that of the others. Furthermore, the paper fibers became shorter, and the number of them increased as the mesh became smaller. It can be concluded that the quantity of paper fibers in UDPCs 100 (sieved with 100–200 mesh) solution was significantly higher than the others. Initially, the paper–plastic complex and microfibers entangled and distributed randomly in the solution. As time went by, the paper fibers became swollen and their profiles became vague after 0.5 h, implying that the dissolution process involved swelling and dissolution, and AmimCl was a powerful solvent for lignocellulose. However, the paper–plastic complex was still clearly presented in the solution for CPE20 and CPE40, which was attributed to the larger size of the ground PE coatings, which prevented the stuck paper fibers from dissolving. The solution states for UDPCs of different sizes displayed by the POM pictures after 3 h and 4 h were similar, indicating that most fibers of UDPCs can be dissolved in AmimCl under mild conditions after 3 h. Additionally, the digital picture of the cellulose/PE solution mixtures after 4 h is shown in Figure 2q. As presented, the solution was opaque and was dark yellow, ascribed to the lignin, ground PE coatings, and other additives in the raw materials. Meanwhile, the viscosity of the CPE100 solution was higher than that of the CPE20 solution due to there being more fibers dissolved in the CPE100 solution. Consequently, UDPCs can be dissolved in AmimCl to achieve a homogeneous and stable mixed solution.

The dissolution mechanism of cellulose in ionic liquids has been studied systematically, and it is known that cellulose is dissolved effectively by the synergistic effect of both anions and cations [45,46]. Nevertheless, more studies need to be conducted to clarify the dissolution mechanism of lignocellulose in ionic liquids [43].

### 3.2. Transparency of Cellulose/PE Composite Films

**Figure 3:**
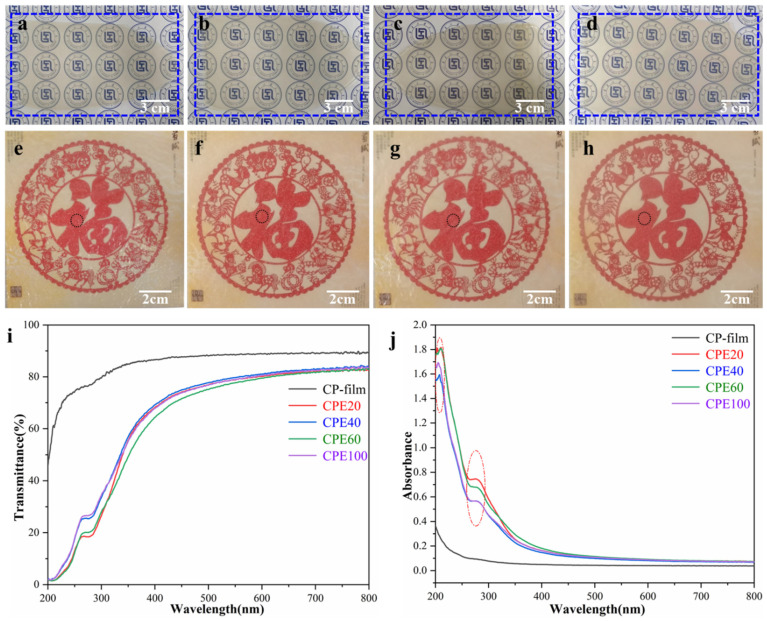
Digital pictures of the cellulose/PE composite gels (**a**) Gel-CPE20; (**b**) Gel-CPE40; (**c**) Gel-CPE60; (**d**) Gel-CPE100) and films (**e**) CPE20; (**f**) CPE40; (**e**,**g**) CPE60; (**h**) CPE100; UV-Vis spectra (**i**,**j**) of CP-film and cellulose/PE composite films.

The transparency of packaging and wrapping materials is important in relation to their application field, and optical photographs and UV-Vis spectra of the cellulose/PE composite films are shown in Figure 3. Generally, the cellulose gels and films prepared from the pure cotton or wood pulp have a colorless transparency. Conversely, it can be seen that the cellulose/PE composite gels (Figure 3a–d) and films (Figure 3e–h) were brown, but they were still in high transmittance. Moreover, the UV-vis curves in the visible region (400–800 nm) can be used to quantifiably describe the difference between the traditional cellulose film (CP-film) and the composite films (CPE20, CPE40, CPE60, and CPE100), in which the transmittance of the CP-film was a little higher than that of the composite films (Figure 3i), because lignin, ground PE coatings, and other additives originating from the UDPCs were still embodied in the composite films. It is worth noting that the white ground PE coatings were obviously exhibited in the composite gels and films due to the insolubility of PE in AmimCl. However, the texture of the cellulose/PE composite is homogeneous, and the differences in transmittance between CPE20, CPE40, CPE60, and CPE100 were minor, indicating that the size of un-dissolved PE pieces showed no obvious effect on the transmittance of the composite films. Additionally, as demonstrated in Figure 3j, the cellulose/PE composite films displayed obvious absorption peaks at around 205 nm and 270 nm and possessed better UV-shielding property than that of the traditional cellulose film (CP-film), because lignin contains numerous phenolic groups and is a kind of natural absorbing ultraviolet light substance [43,47]. Consequently, the cellulose/PE composite films fabricated from the bamboo-based disposable paper cups by the “one-pot method” showed good transmittance in the visible region and UV-shielding properties, which is favorable for their uses as packaging and wrapping materials.

### 3.3. Structure and Crystallinity

**Figure 4:**
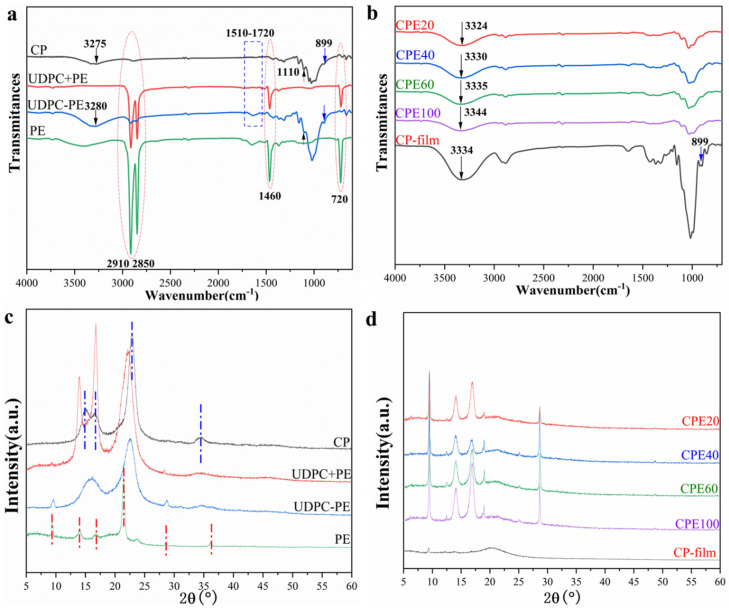
(**a**) XRD of CP, UDPC+PE, UDPC-PE and commercial PE film; (**b**) XRD of CP-film and the cellulose/polyethylene composite films; (**c**) FTIR of CP, UDPC+PE, UDPC-PE and commercial PE film; (**d**) FTIR of CP-film and the cellulose/polyethylene composite films.

Both X-ray diffraction and ATR-FTIR were carried out to investigate the crystallization, components, and structural changes of the cellulose/PE composite films and raw materials. Detailed information of the components and crystalline phase changes of CPs, UDPCs (the PE side and after peeling off the PE coatings), PE film, and cellulose/polyethylene composite films are clarified by the X-ray diffraction patterns in Figure 4a,b. It is clear that the commercial PE film showed obvious peaks at approximately 2*θ* = 9.5°, 14.0°, 16.8°, 21.5°, 23.8°, 28.6°, and 36.2° [44], which can be used as reference. Generally, the raw materials of cellulose possessed typical cellulose I, which exhibited peaks at approximately 2*θ* = 15.1°, 16.8°, 22.8°, and 34.5°, corresponding to (1–10), (110), (200), and (004) crystal planes [27,32]. Hence, all the raw material CP, UDPC+PE (the PE coatings side), and UDPC-PE (after peeling off PE coatings) demonstrated prominent peaks of cellulose I (Figure 4a). However, compared with CP, the peaks of UDPC+PE (the PE coatings side) at 2*θ* = 15.1° and 22.8° shifted to the lower degree, due to the existence of the PE coatings. Meanwhile, the peaks of UDPC-PE (after peeling off PE coatings) at 2*θ* = 22.8° repositioned and 2*θ* = 15.1° and 16.8° merged, implying that most PE coatings can be removed by the peeling process. Additionally, removing PE coatings completely and thoroughly is impossible, because the melted PE liquid easily permeates into the paper board voids during the production process of paper cups. Consequently, recycling or reusing the UDPCs is a challenging task in the industry, and the “one-pot method” in this work is therefore feasible and meaningful. It is worth noting that the cellulose phase is usually transformed from I to II after the dissolution and regeneration. Herein, the regenerated cellulose film (CP-film) and the cellulose/polyethylene composite films displayed obviously different patterns from those of the raw materials. Meanwhile, all the composite films demonstrated obvious peaks at 9.5°, 14.0°, 21.5°, and 28.6°, which were attributed to the peaks of the PE coatings, combined with the fact that the PE coatings were still confined in the composite films. Compared with the raw materials, the densities of CP-film and cellulose/polyethylene composite films decreased sharply, meaning that the cellulose crystallinity index decreased prominently after the dissolution and regeneration processes. Thus, the characteristic peaks of PE coatings were obviously enhanced. It is worth noting that CPE20, CPE40, CPE60, and CPE100 exhibited similar profiles, suggesting that the size of the PE pieces had no obvious influence on the crystalline structure of cellulose films, which is consistent with the Ultraviolet and Visible (UV-Vis) spectra results.

FTIR can provide information on the components and structures of the raw materials and the products. Figure 4c,d present the spectra of CPs, UDPCs, commercial PE film, and cellulose/polyethylene composite films. It can be concluded that CPs, UDPCs (the PE side and after peeling off the PE coatings), CP-films, and cellulose/polyethylene composite films showed similar spectra, indicating that the main component in the raw materials and the products was cellulose and no chemical reactions occurred between AmimCl and cellulose during the UDPCs dissolution and regeneration processes. In other words, AmimCl was the non-derivative solvent for UDPCs. However, UDPC+PE (the PE coatings side) displayed similar peaks at approximately 2910 cm^−1^, 2850 cm^−1^, 1460 cm^−1^, and 720 cm^−1^ to those of commercial PE film, which was obviously different from those of CP and UDPC-PE (after peeling off PE coatings), suggesting that there was a dense and homogeneous PE coating inside the paper cups to prevent liquid leakage, and most PE coatings can be removed from paper board by a simple peeling process. Additionally, compared to CP, the UDPC-PE (after peeling off PE coatings) exhibited broad peaks that ranged from 1720 cm^−1^ to 1510 cm^−1^, due to the existence of lignin and hemicellulose [32,48]. Generally, the O-H stretching band, ranging from 3200 to 3700 cm^−1^, and the C-H stretching band, ranging from 2700 to 3000 cm^−1^, are sensitive to the structure change of cellulose [27,32]. It can be seen that the CP and UDPC-PE demonstrate O-H stretching peaks at 3275 cm^−1^ and 3280 cm^−1^, respectively. However, these peaks exhibited a prominent blue shift and moved to 3334 cm^−1^ for CP-film and 3324 cm^−1^, 3330 cm^−1^, 3335 cm^−1^, and 3344 cm^−1^ for cellulose/polyethylene composite films. It was a little difficult to use the C-H stretching peak (2700–3000 cm^−1^) for analysis because it merged with those of PE. Additionally, the absorption peaks at 899 cm^−1^ and 1110 cm^−1^ are also used to characterize the changes of cellulose structure [32,49]. As shown in Figure 4d, CPs and UDPCs had an obvious 1110 cm^−1^ absorption peak, while it weakened in the CP-film and the cellulose/polyethylene composite films. Meanwhile, the peak at 899 cm^−1^ was not prominent in the spectra of CPs and UDPCs (the PE coatings side and after peeling off PE coatings), but it was weakly enhanced in the CP-film and cellulose/polyethylene composite films. Additionally, the spectra of the composite films prepared directly from UDPCs in different sizes are almost the same. These results suggest that the hydrogen bonding or crystal structure changed significantly after the dissolution and regeneration of UDPCs, and the size of ground PE showed no prominent impact on the crystal structure of films, which was also in correspondence with former results.

### 3.4. Mechanical Property, Hydrophilicity and Thermal Degradation

**Figure 5:**
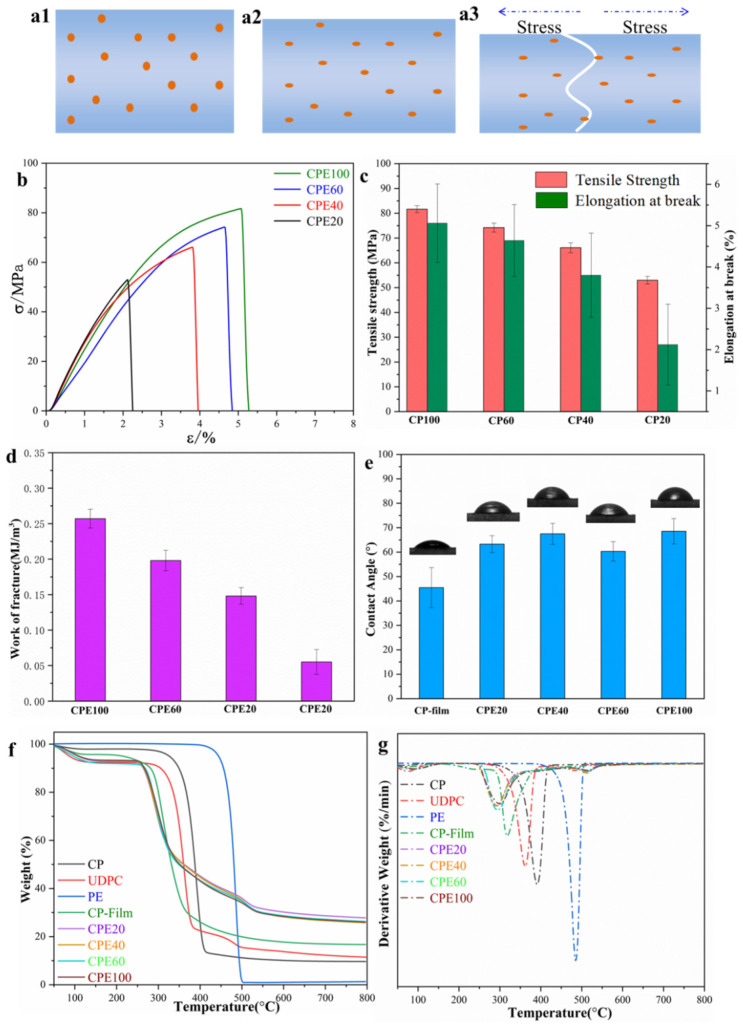
The stretching process of the cellulose/polyethylene composite films (**a1**–**a3**), stretch strain-stress (**b**), tensile strength and elongation at break (**c**), work of fracture of the cellulose/polyethylene composite films (**d**), the water contact angles of CP-film and the cellulose/polyethylene composite films (**e**), TG curves (**f**), and DTG curves (**g**) of CP, UDPC, commercial PE film, CP-film, and the cellulose/polyethylene composite films.

It is known that mechanical properties are a decisive index for films employed as packaging or wrapping materials, and the degree of polymerization (DP) directly affects the tensile strengths of polymer films [32,44]. As measured by Ubbelodhe viscometry, the DP of cellulose in bamboo-based UDPCs was approximately 304, suggesting that the cellulose/PE composite films possessed a relatively high tensile strength. As displayed in Figure 5(a1–a3), under stretching, PE particles and the polymer molecular chains were stretched along the direction of stretching (Figure 5(a2)). Eventually, the cellulose/PE composite films fractured at the interfacial defect between the cellulose matrix and the PE particles (Figure 5(a3)). In fact, the cellulose molecules and PE molecules were difficult to blend due to their different molecular structures. Generally, pre-treatments are necessary before the two different components are mixed together to fabricate composites. However, in this work, cellulose/PE composite film with a uniform structure could be achieved by the “one-pot method” without any pretreatment. Differently from previous works, where the natural fibers were usually used as the reinforced phase [34,35,36,37], in this work the bamboo-based paper board was dissolved and regenerated into a cellulose film matrix, and the undissolved PE pieces were used as the additives. Hence, transparent cellulose/polyethylene composite films were achieved. Figure 5b–d demonstrates the stress-strain curves, the average tensile strength, the elongation at break, and the work of fracture of the composite films. The tensile strength, elongation at break, and the work of fracture of CPE100 (sieved with 100–200 mesh) were approximately 82 Mpa, 5.1%, and 0.257 MJ/m^3^, respectively, while the tensile strength, the elongation at break, and the work of fracture of the composite films decreased with the increasing size of the PE coatings, resulting from the decreased interfacial adhesion. The tensile strength and elongation at break of CPE60, CPE40, and CPE20 were approximately 74 Mpa, 66 Mpa, 53 Mpa, and 4.6%, 3.8%, 2.1%, respectively, while the work of fracture of the composite films were approximately 0.198 MJ/m^3^, 0.148 MJ/m^3^, and 0.055 MJ/m^3^, respectively. Nevertheless, compared to traditional commercial PE film (9–12 MPa) [42,43], the cellulose/PE composite films possessed higher tensile strength, showing a great potential to be used in the packaging or wrapping fields. Additionally, it is worth noting that the elongation at break of the composite films is also below 5%, which is a characteristic of rigid backbone cellulose films, indicating the low content of PE coatings in the composite films [25,27]. Fortunately, it was found that adding plasticizers can improve the elongation at break of cellulose-based films, and glycerol was used in our previous work, where the elongation at break effectively could be improved 5–10 times by adding 8% glycerol plasticizers [27,42]. Thus, the cellulose/PE composite films made from bamboo-based disposable paper cups showed good mechanical properties and can be used as packaging and wrapping materials.

Generally, cellulose films are hydrophilic due to copious inter-molecular and intra-molecular hydrogen bonds, limiting their uses as packaging or wrapping materials [43]. As shown in Figure 5e, the water contact angles (WCAs) of pure cellulose film and CP-film were around 45.6°, meaning that cellulose films exhibited good hydrophilicity. However, compared to CP-film, the composite films possessed higher WCAs, 63.3°, 67.5°, 60.3°, and 68.6° for CPE20, CPE40, CPE60, and CPE100, respectively, which is attributed to hydrophobic lignin, PE coatings, or other additives contained in UDPCs and composite films. Meanwhile, the composite films showed a much rougher surface than that of neat cellulose film. Hence, the cellulose/PE composite films fabricated from bamboo-based disposable paper cups demonstrated their superiority in the packaging and wrapping fields due to their relatively good hydrophobicity.

Thermal performance is also an important index to judge the quality of materials. Both the thermogram curves (TG) and differential thermogram (DTG) curves of CP, UDPC, commercial PE film, and cellulose/PE composite films were recorded to investigate their thermal stability. As exhibited in Figure 5f,g, the minor mass loss at temperatures below 200 °C was ascribed to the moisture loss [43]. It can be concluded that the hydrophilic cellulose materials contained more water than that of the hydrophobic PE film. Compared to other cellulose materials, the CP displayed the highest temperature of maximum weight loss rates (T_max_) at 395 °C and onset decomposition temperature (T_onset_) at 280 °C, because the raw material CP possessed the highest DP and degree of crystallinity. Generally, the thermal properties of the regenerated cellulose-based films declined, due to the decrease of DP and degree of cellulose crystallinity after the dissolving and regeneration processes [32,50]. Herein, the T_max_ of the regenerated cellulose film CP-film was 320 °C. Meanwhile, the T_max_ of UDPCs was 360 °C, higher than those of cellulose/PE composite films. It is worth noting that UDPCs and the composite films displayed minor peaks at approximately 485 °C, attributed to the T_max_ of the PE coatings, which are a common phenomena of physically mixed composites. Moreover, the T_max_ of the commercial PE film was also approximately 485 °C, suggesting that the ground PE coatings still possessed good thermal stability after the regeneration process. Additionally, the residue weights of composite films were bigger than that of the CP-film, which is ascribed to the impurities confined in the composite films. In short, the cellulose/PE composite films had thermal properties that were good enough to meet industry demands.

### 3.5. Morphology of Cellulose/Polyethylene Composite Films

**Figure 6:**
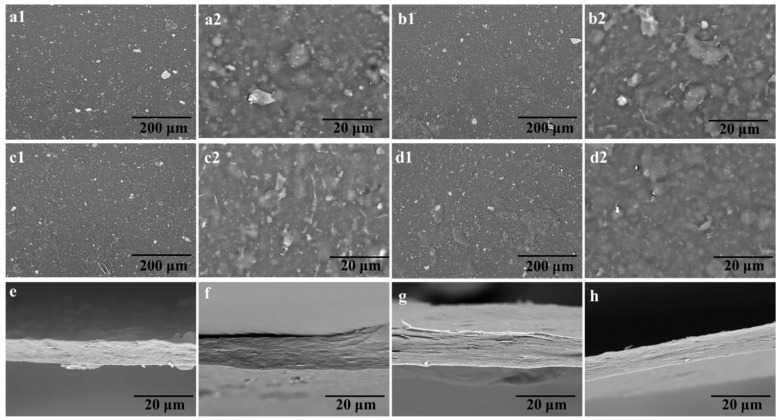
(**a1**–**h**) SEM micrographs of the cellulose/PE composite films, (**a1**–**d2**) surface images of the cellulose/PE composite films with different magnification, (**e**–**h**) cross section images of the cellulose/PE composite films.

To acquire more information regarding the structures of cellulose/PE composite films, SEM micrograph were recorded. Figure 6(a1–h) displays the surface and cross section morphology of the cellulose composites. It can be seen that all composites possess a relatively flat surface, although lignin and PE pieces are still confined in the films (Figure 6(a1–d2)). However, the ground PE coatings are obvious on the surface of films, and the size of them declined with the increased number of the screen mesh, leading to a more uniform and homogeneous structure (CPE100, Figure 6(d1,d2)). Meanwhile, the composite films demonstrated a loose inner texture due to the additives, but CPE100 possessed the densest and most homogeneous cross section structure due to the smallest size of impurities, leading to the highest tensile strength of CPE100.

## 4. Conclusions

By using 1-allyl-3-methylimidazolium chloride (AmimCl) as a solvent, transparent and uniform cellulose/polyethylene composite films were obtained from used bamboo-based disposable paper cups through a “one-pot method” without any pretreatment, where the dissolved lignocellulose was employed as the matrix and the un-dissolved PE coatings were used as the additives. Compared to traditional pure cellulose films, it was found that the composite films displayed a good UV-shielding property and hydrophobicity, showing superiority as packaging or wrapping materials. Meanwhile, cellulose changed from I to II after the dissolution and regeneration processes, and the thermal property of the regenerated cellulose-based materials decreased significantly. Nevertheless, the composite film still exhibited competitive thermal properties. Additionally, the size of the ground PE coatings showed obvious impacts on the structures and properties of the composite films. Owing to the strongest adhesion between the cellulose matrix and the ground PE coatings, the CPE100 possessed the smoothest, most compact and homogenous texture and the best mechanical properties: tensile strength, elongation at break, and work of fracture were approximately 82 Mpa, 5.1%, and 0.257 MJ/m^3^, respectively. Generally, it is difficult to recycle and reuse used disposable paper cups (UDPCs) in industry, because the non-degradable inner PE coatings and the degradable paper board are bonded tightly, and separating them economically, completely, and efficiently is impossible. In this work, a facile and green “one-pot method” to realize the complete use of bamboo-based disposable paper cups by fabricating cellulose/polyethylene composite films was developed, which is significant in terms of environmental protection and the maximum utilization of natural resources.

## Data Availability

The data presented in this study are available on request from the author.
